# Cost-effectiveness of multidisciplinary care for patients with chronic kidney disease in Japan

**DOI:** 10.1007/s10157-026-02883-0

**Published:** 2026-05-18

**Authors:** Hiromu Tomishima, Motoki Odawara, Tsuguru Hatta, Yoshihiko Imamura, Tsutomu Sakurada, Takashi Maruyama, Masanori Abe, Shinya Kaname, Masaomi Nangaku, Hiroshi Nishi

**Affiliations:** 1 Faculty of Medicine, The University of Tokyo, Tokyo, Japan; 2 Division of Nephrology and Endocrinology, The University of Tokyo Graduate School of Medicine, 7-3-1 Hongo, Bunkyo-ku, Tokyo, 113-8655 Japan; 3 Department of Medicine, Hatta Medical Clinic, Kyoto, Japan; 4 Department of Nephrology, Nissan Tamagawa Hospital, Tokyo, Japan; 5 Division of Nephrology and Hypertension, Department of Internal Medicine, St. Marianna University School of Medicine, Kanagawa, Japan; 6 Division of Nephrology, Hypertension and Endocrinology, Department of Medicine, Nihon University School of Medicine, Tokyo, Japan; 7 Department of Nephrology and Rheumatology, Kyorin University School of Medicine, Tokyo, Japan; 8 Department of Internal Medicine, Kichijoji Asahi Hospital, Tokyo, Japan

**Keywords:** Chronic kidney disease, Cost-effectiveness, Multidisciplinary care

## Abstract

**Background:**

Multidisciplinary care (MDC), where a team of various health professionals provides guidance to patients, is an effective approach for chronic kidney disease (CKD) and has been reimbursed by Japanese public healthcare insurance in 2024. To sustainably achieve medical benefits with limited resources, we examined the cost-effectiveness of MDC in Japan.

**Methods:**

A Markov model from a healthcare system perspective was used to evaluate the cost-effectiveness of adding MDC to the standard treatment for CKD in Japan over lifetime. Each cohort with an initial CKD stage (G3a, G3b, and G4) was analyzed. The parameters were based on previous studies including Chronic Kidney Disease Japan Cohort study. An incremental cost-effectiveness ratio (ICER) of < 5,000,000 Japanese yen (JPY) per quality-adjusted life-year (QALY) was considered cost-effective. One-way deterministic analyses, probabilistic sensitivity analyses, and scenario analyses were conducted to ensure the robustness of the results.

**Results:**

Adding MDC to the standard treatment for CKD decreased cost by 10.66 million, 8.42 million, 4.21 million JPY and increased utility by 9.52, 8.70, 7.17 QALYs in CKD G3a, G3b, and G4 cohort, respectively, suggesting that it was a dominant strategy. Sensitivity analyses showed that adding MDC was cost-effective in more than 99% of cases in all cohorts with an ICER threshold of 5,000,000 JPY. Scenario analyses, where smaller therapeutic effects of MDC were estimated, showed compatible results.

**Conclusion:**

Based on our model, adding MDC to the standard treatment for CKD stages G3a, G3b, and G4, is highly likely to be a cost-effective strategy in Japanese healthcare system.

## Introduction

Chronic kidney disease (CKD) is a growing healthcare problem worldwide because of its high prevalence and mortality [1]. The all-age prevalence of CKD exhibited a 29.3% increase from 1990 to 2017, and the number of patients with CKD has exceeded 700 million in 2017 [2]. The rising prevalence of CKD and the increasing complexity of CKD patients are driving up the costs of CKD care, imposing a significant economic burden [1]. In a previous study, the annual cost of CKD treatment in 31 countries was estimated to increase by 9.3% (from $372.0 billion to $406.7 billion) between 2022 and 2027 [3]. The study also assumed that annual cost associated with kidney replacement therapy (KRT) would increase by 10.0% (from $169.6 billion to $186.6 billion) in the meantime, and patients receiving KRT would constitute 5.3% of the CKD population but contribute 45.9% of the total costs by 2027 [3]. Therefore, not only it is essential to take proactive approaches to address CKD, but such approaches must also be acceptable from an economic point of view.

In addition to conventional pharmacological therapy with renin-angiotensin system inhibitors (RASi) and sodium-glucose cotransporter-2 inhibitors (SGLT2i), nonsteroidal mineralocorticoid receptor antagonists have recently been shown to be effective for the treatment of diabetic kidney diseases in delaying its progression [4–8]. Furthermore, cost-effectiveness of RASi and SGLT2i have been demonstrated [9, 10]. Importantly, in addition to such established pharmacological therapies, the delivery of multidisciplinary care (MDC) is also recommended for the care of patients with CKD. Importantly, in addition to pharmacological therapy, the delivery of multidisciplinary care (MDC) is also recommended for the care of patients with CKD [4]. MDC teams typically consist of nephrologists, nurses, dietitians, social workers, and pharmacists [11]. They provide dietary counseling, medication reconciliation, education on KRT modality selection, vascular access planning, and transplant workup [12, 13]. In Japan, the Kidney Disease Care Educator program was initiated in 2017 by the Japan Kidney Association. The program’s objectives are to prevent the progression of CKD and to enhance the patients’ quality of life (QOL). Nurses, registered dietitians, and pharmacists can obtain the qualification to participate in the program by meeting specific requirements. Certified professionals have accurate knowledge about CKD and its care, can provide comprehensive guidance on lifestyle modification and dietary and medication counseling, and offer explanation about KRT. This program has made significant contributions to MDC in Japan. A recent clinical study in Japan showed that MDC for CKD patients significantly suppressed the decline in estimated glomerular filtration rate (eGFR) [14]. This nationwide, multicenter, retrospective study provided MDC to 3015 Japanese patients with CKD stage G3a to G5 and evaluated decrease in eGFR before and after intervention.

Under Japanese healthcare system, MDC for CKD has been reimbursed by public healthcare insurance as a means of preventing dialysis since 2024. A guidance by doctor(s) together with medicals staffs such as nurses or dieticians is required for its monthly reimbursement [15]. Although some studies reported that MDC is cost-effective for treating patients with CKD in other countries [16–19], whether it is also the case in Japan remains unclear. Therefore, the objective of this study was to evaluate the cost-effectiveness of adding MDC to standard treatment for CKD in Japan.

## Methods

### Model

**Fig. 1:**
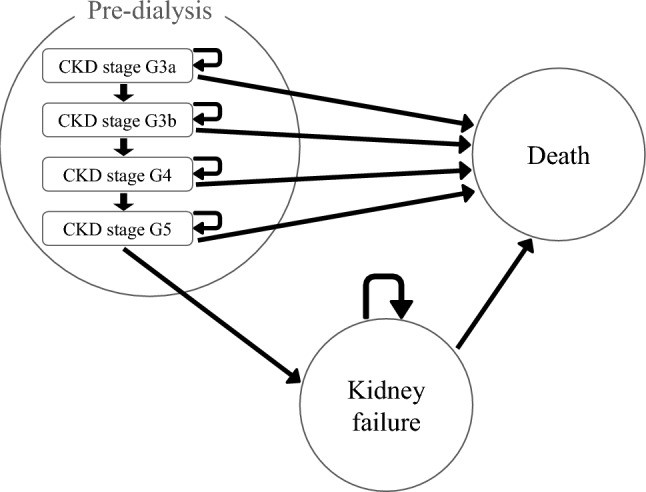
Design of Markov model. Markov model for cost-effectiveness analysis of multidisciplinary care in treating CKD. The model applied to each annual cycle. Each patient was in one of the following three health statuses: pre-dialysis, kidney failure, and death. Pre-dialysis status was further classified into four stages according to eGFR. Each year in the pre-dialysis status, patients’ eGFR declined at the designed rate. Each year, patients moved to another state in the direction of arrows if the conditions were met. All patients started with the CKD stage designated by their cohorts

We used a Markov model to evaluate the cost-effectiveness of adding MDC for patients with CKD from a perspective of Japanese healthcare system. The cost and effectiveness of treatments were calculated among two groups, standard treatment group (control group) and standard treatment plus MDC group (MDC group), over a lifetime period. Three health statuses were defined: pre-dialysis, kidney failure (KF) and death (Fig. 1). Pre-dialysis status was further divided into four CKD stages (G3a, G3b, G4, and G5) according to eGFR as shown below.

- CKD stage G3a: 45 ≦ eGFR < 60 ml/min per 1.73 m^2^
- CKD stage G3b: 30 ≦ eGFR < 45 ml/min per 1.73 m^2^
- CKD stage G4: 15 ≦ eGFR < 30 ml/min per 1.73 m^2^
- CKD stage G5: eGFR < 15 ml/min per 1.73 m^2^

KF status was defined as a condition requiring KRT, which was started immediately when the patient entered the state. Patients in the pre-dialysis status transitioned to the KF or death statuses when corresponding events occurred. Patients in the KF status transitioned to the death status when they died.

We performed 5000 simulations over 120 cycles, with each cycle representing 1 year, to evaluate life-time cost and effectiveness and calculated their mean values in the two groups. The previous cost-effectiveness analyses in relation to CKD showed different results among a disease grade-based model and an eGFR-based microsimulation [20]. In this study, eGFR-based microsimulations were used, in which each patient had their own eGFR. The variances of event probabilities, costs, and effectiveness depended on the CKD stage in the pre-dialysis status.

In the model, we applied the effects of MDC, which were shown to suppress eGFR decline in the previous study [14]. This previous study was a multicenter, retrospective, observational study that followed 3015 Japanese patients with CKD stages G3–G5 for 3 years. The event probabilities were based on the Chronic Kidney Disease Japan Cohort (CKD-JAC) study, a multicenter prospective cohort study of 2966 patients aged 20–75 years with CKD stage G3–G5 over 4 years [21–23].

We analyzed three cohorts (CKD stages G3a, G3b, and G4) with initial eGFR values within the designed ranges. All patients in each cohort began in the pre-dialysis status and transitioned to the KF status when their eGFR fell below the designed threshold, or to the death status when the event occurred. The results were calculated as incremental cost-effectiveness ratio (ICER). The willingness to pay (WTP) threshold was set at 5 million Japanese yen (JPY) per quality-adjusted life-year (QALY), consistent with the previous reports [24–26]. All analyses were performed using TreeAge Pro Healthcare Version 2025.

### Population and intervention

The patients’ backgrounds were based on the CKD-JAC study [27], since the event rates were referred from the study as described below. The average age was 60.3 years, and 62% of the patients were male. Both treatment groups received standard treatment in Japan based on the guideline at the time of the background studies [14, 27], which included RASi but not SGLT2i [28]. Those in the MDC group received additional MDC care while in the pre-dialysis status. According to the previous study [14], multidisciplinary care was defined as follows: (1) care by a team comprising nephrologists and professionals from other disciplines, including nurses, registered dietitians, pharmacists, physical therapists, social workers, clinical engineers, and clinical laboratory technicians, and (2) an operational model of multidisciplinary care, whereby patients with CKD were managed medically, received patient education, and were encouraged to make lifestyle modifications according to the stage of CKD. The quality of the educational content provided was maintained in accordance with the recommendations of the Japanese Society of Nephrology, Japanese Society for Dialysis Therapy, Japan Society for Transplant, and Japanese Society for Clinical Renal Transplantation or the CKD Teaching Guidebook for Certified Kidney Disease Educators by the Japan Kidney Association.

### Initial values, decline of eGFR and threshold for kidney failure

The initial eGFR value was randomly assigned to each patient in the uniform distribution among the designated range of the initial CKD stage in each cohort.

The annual eGFR decline in the control group (4.05, 6.20, and 8.43 ml/min per 1.73 m^2^ per year) depended on CKD stage (G3, G4, and G5) based on the previous study and was applied to each patient’s eGFR value every cycle [14]. The respective values of the decline in the MDC group were 1.82, 1.33, and 0.72 at CKD stages G3, G4, and G5 [14]. The annual declines in the control group were based on the 12-month eGFR change before the MDC intervention, and the declines in the MDC group were based on the change in 12 months after the MDC intervention started. When eGFR fell below the thresholds of 45, 30, or 15 ml/min per 1.73 m^2^, patients progressed to CKD stage G3b, G4, or G5, respectively.

The patients moved to the KF status when their eGFR became lower than the threshold value (6.52 ml/min per 1.73 m^2^), which was referred from a previous Japanese study [29]. Table 1 presents the values related to eGFR.

**Table 1:** Variables in the model

Parameter	Value	Range for one-way sensitivity analysis	Distribution for probabilistic sensitivity analysis (mean ± SD, type of distribution)	References
Annual decline of eGFR				
MDC				
CKD stage G3a/3b	1.82	1.365–2.275	1.82 ± 0.2275, γ	[14]
CKD stage G4	1.33	0.9975–1.6625	1.33 ± 0.16625, γ	
CKD stage G5	0.72	0.54–0.9	0.72 ± 0.09, γ	
Standard treatment				
CKD stage G3a/3b	4.05	3.0375–5.0625	4.05 ± 0.50625, γ	
CKD stage G4	6.20	4.65–7.75	6.20 ± 0.775, γ	
CKD stage G5	8.43	6.3225–10.5375	8.43 ± 1.05375, γ	
Threshold of eGFR for kidney failure (ml/min per 1.73 m^2^)	6.52	0–14.92	6.52 ± 4.20, γ	[28]
Probability of death				
CKD stage G3a	0.0036	0.0027–0.0045	0.0036 ± 0.00045, β	[23]
CKD stage G3b	0.0064	0.0048–0.008	0.0064 ± 0.0008, β	
CKD stage G4	0.009	0.0068–0.0113	0.009 ± 0.001125, β	
CKD stage G5	0.0077	0.0058–0.0096	0.0077 ± 0.0009625, β	
Kidney failure	0.097	0.0728–0.1213	0.097 ± 0.012125, β	[29]
Cost				
CKD stage G3a/3b	¥337,000 ($2254)	¥168,500–505,500	¥337,000 ± 84,250, γ	[30]
CKD stage G4	¥793,000 ($5304)	¥396,500–1,189,500	¥793,000 ± 198,250, γ	
CKD stage G5	¥988,000 ($6608)	¥494,000–1,482,000	¥988,000 ± 247,000, γ	
Kidney failure	¥6,000,000 ($40,132)	¥3,000,000–9,000,000	¥6,000,000 ± 1,500,000, γ	[31]
MDC	¥30,000 ($201)	¥15,000–45,000	¥30,000 ± 7500, γ	[15]
Utility				
CKD stage G3a/3b	0.883	0.857–0.909	0.883 ± 0.110375, β	[32]
CKD stage G4	0.839	0.794–0.884	0.839 ± 0.104875, β	
CKD stage G5	0.798	0.757–0.839	0.798 ± 0.09975, β	
Kidney failure	0.79	0.5925–0.9875	0.79 ± 0.09875, β	[33, 34]

*CKD* chronic kidney disease, *MDC* multidisciplinary care, *eGFR* estimated glomerular filtration rate

### Events

Patients moved to the death status based on the probabilities shown in Table 1. In the pre-dialysis status, the probabilities depended on their CKD stages (G3a, G3b, G4, or G5), according to the data from the CKD-JAC study [23]. In the KF status, the probability was based on the previous study [30]. Since the previous study did not include data on contribution of MDC to CKD mortality, the event rate values were the same for both groups.

### Costs

Table 1 presents the data of cost. The costs of the treatments per cycle were calculated based on the patient’s status in terms of the Japanese healthcare system. In the pre-dialysis status, outpatient costs were taken into consideration [31]. Annual MDC costs were calculated at 36,000 JPY in the first year (3000 JPY per month multiplied by 12 months) and 30,000 JPY thereafter (2500 JPY per month multiplied by 12 months) [15]. In the KF status, the cost was calculated by averaging the cost of each KRT type (hemodialysis, peritoneal dialysis, and kidney transplant), weighted by the proportion of patients in Japan receiving each therapy [32]. All costs were discounted at 2% per year based on the Japanese guidelines [25].

### Utility

QALYs based on the EuroQol 5-Dimension Questionnaire were used to measure the effects of each treatment. In the pre-dialysis status, QALY per cycle was based on CKD stages (G3, G4, and G5) according to the previous report [33]. In the KF status, QALY per year was calculated by averaging the QOL of patients in each KRT modality (hemodialysis, peritoneal dialysis, and kidney transplant) [34, 35], weighted by the proportion of patients in Japan [32]. Since the previous study did not include data on how MDC contributed to the QOL of CKD patients, the QOL values were identical for both groups. Table 1 presents the QOL values. All utilities were discounted at 2% per year based on the Japanese guidelines [25].

### Sensitivity analysis

We conducted sensitivity analyses to evaluate the robustness of our results. One-way deterministic analyses were conducted to check the effects of all the variables, and the results were summarized into tornado diagrams. We also performed probabilistic sensitivity analyses to examine the effects of parameter uncertainty. We created probability distributions for all the variables and obtained 500 sets of randomly sampled variables. A total of 5000 microsimulations per set were conducted, and the results were plotted. The threshold for the willingness to pay was set at 5 million JPY per QALY.

For the one-way deterministic analyses, the value ranges were either ± 2 × standard deviation (SD) or 95% confidence intervals if reported or otherwise ± 25% for probabilities and utilities, and ± 50% for costs and estimated values. For the probabilistic sensitivity analyses, SDs were either reported values or 1/8 of the mean values for probabilities and utilities and 1/4 of the mean values for costs and estimated values.

### Scenario analysis

Scenario analysis was performed to assess the validity of the base-case analysis. We evaluated the cost-effectiveness of MDC based on an assumption that its effect of suppressing eGFR decline was smaller than reported, using higher eGFR decline rate for the MDC group [14, 36]. The referred values were from CKD-JAC study, where MDC was not practiced in particular but the patients were adequately managed under specialized nephrologists. The annual eGFR declines in the study were 1.925, 2.056, 3.182, and 3.754 at CKD stages G3a, G3b, G4, and G5, respectively, which were larger than the values for the MDC group in the base-case analysis but smaller than those for the control group [36].

## Results

### Base-case analysis

The results of the base-case analysis are presented in Table 2. In the CKD stage G3a cohort, the total costs in MDC and standard treatment groups were 34.84 million JPY and 45.51 million JPY (233.0 thousand United States dollar (USD) and 304.4 thousand USD), respectively, and the utilities were 22.44 QALYs and 12.92 QALYs, respectively. In the CKD stage G3b cohort, the total costs in MDC and standard treatment groups were 39.88 million JPY and 48.30 million JPY (266.7 thousand USD and 323.1 thousand USD), respectively, and the utilities were 19.35 QALYs and 10.66 QALYs, respectively. In the CKD stage G4 cohort, the total costs of MDC and standard treatment groups were 45.96 million JPY and 50.16 million JPY (307.4 thousand USD and 335.5 thousand USD), respectively, and the utilities were 15.69 QALYs and 8.52 QALYs, respectively. Mean KRT-free survival time was increased in MDC group by 22.4, 18.5, 13.5 years in CKD stage G3a, G3b, and G4 cohort, respectively. The addition of MDC to standard treatment reduced the costs by 10.66 million JPY, 8.42 million JPY, and 4.21 million JPY (71.37 thousand USD, 56.32 thousand USD, and 28.16 thousand USD) and increased QALYs by 9.52, 8.70, and 7.17 in CKD stages G3a, G3b, and G4, respectively. These findings indicated that adding MDC was a dominant treatment compared to standard treatment in all analyzed CKD stages.

**Table 2:** Base-case analysis

Cohort	Cost (JPY/USD)	QALY	ICER
CKD stage G3a			
MDC	¥34.84 million/$233.0 thousand	22.44	MDC dominant
Standard treatment	¥45.51 million/$304.4 thousand	12.92	
CKD stage G3b			
MDC	¥39.88 million/$266.7 thousand	19.35	MDC dominant
Standard treatment	¥48.30 million/$323.1 thousand	10.66	
CKD stage G4			
MDC	¥45.96 million/$307.4 thousand	15.69	MDC dominant
Standard treatment	¥50.16 million/$335.5 thousand	8.52	

*CKD* chronic kidney disease, *MDC* multidisciplinary care, *QALY* quality-adjusted life-year, *ICER* incremental cost-effectiveness ratio, *JPY* Japanese yen, *USD* United States dollar

### One-way deterministic analysis

**Fig. 2:**
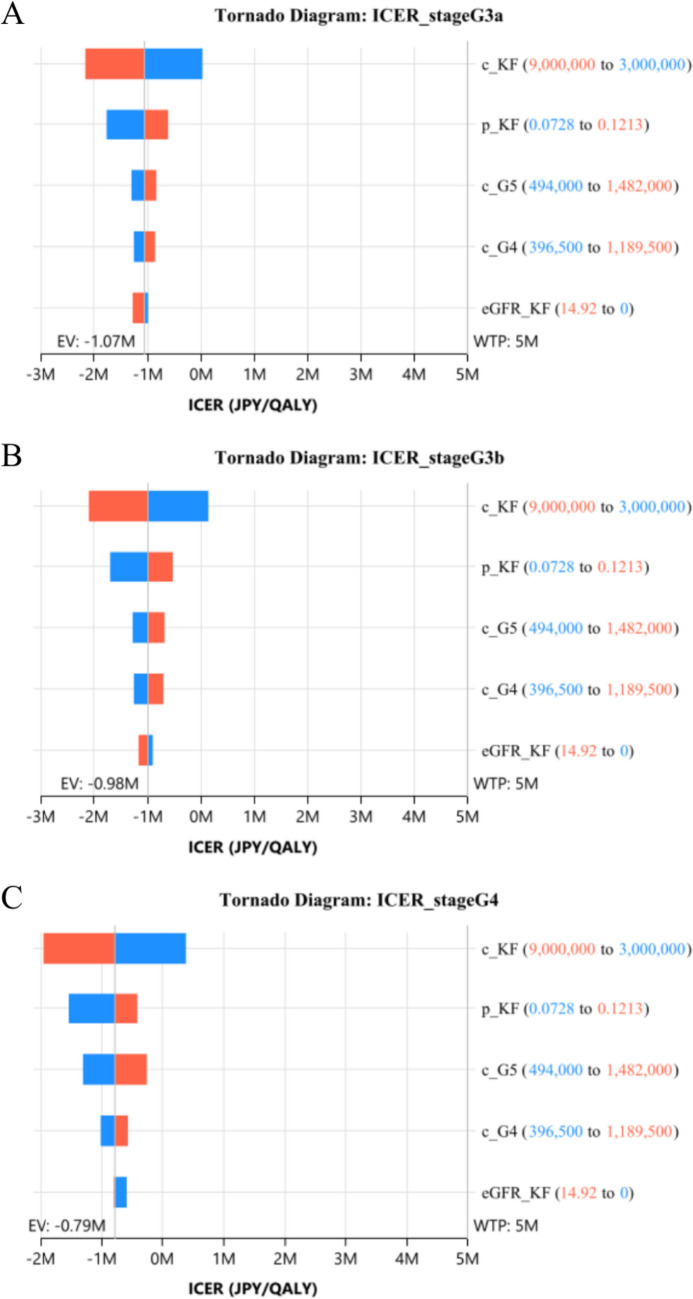
One-way deterministic analysis. Tornado diagrams showing results from one-way deterministic analysis. The vertical axes show five variables which had the greatest impact on the ICER in each cohort. The horizontal axes show the change in the ICER value. Each variable was changed within the designed range. The bars indicate the ranges of ICER. The orange texts and bars correspond to values larger than the base-case analysis, and the blue texts and bars vice versa. Details of each variable are summarized in Table 1. **A**–**C** Patients who were initially at CKD stages G3a, G3b, and G4, respectively. c_G4, c_G5, and c_KF, annual cost for treating patients with CKD stages G4, G5, and kidney failure, respectively; p_KF, probability of death in patients with kidney failure; eGFR_KF, threshold of eGFR for entering the kidney failure status; ICER, incremental cost-effectiveness ratio; QALY, quality-adjusted life year; JPY, Japanese yen; EV, estimated value; WTP, willingness to pay

The results of one-way deterministic analyses are presented in Fig. 2. In all analyzed CKD stage cohorts, the annual cost of kidney failure had the greatest impact on the ICER. The following variables that strongly affected ICER were mortality in patients with kidney failure, annual costs for treating patients with CKD stages G5 and G4, and the threshold value of eGFR for initiating KRT in all the cohorts. No variable was associated with ICER beyond 5 million JPY per QALY in any CKD stage cohort.

### Probabilistic sensitivity analysis

**Fig. 3:**
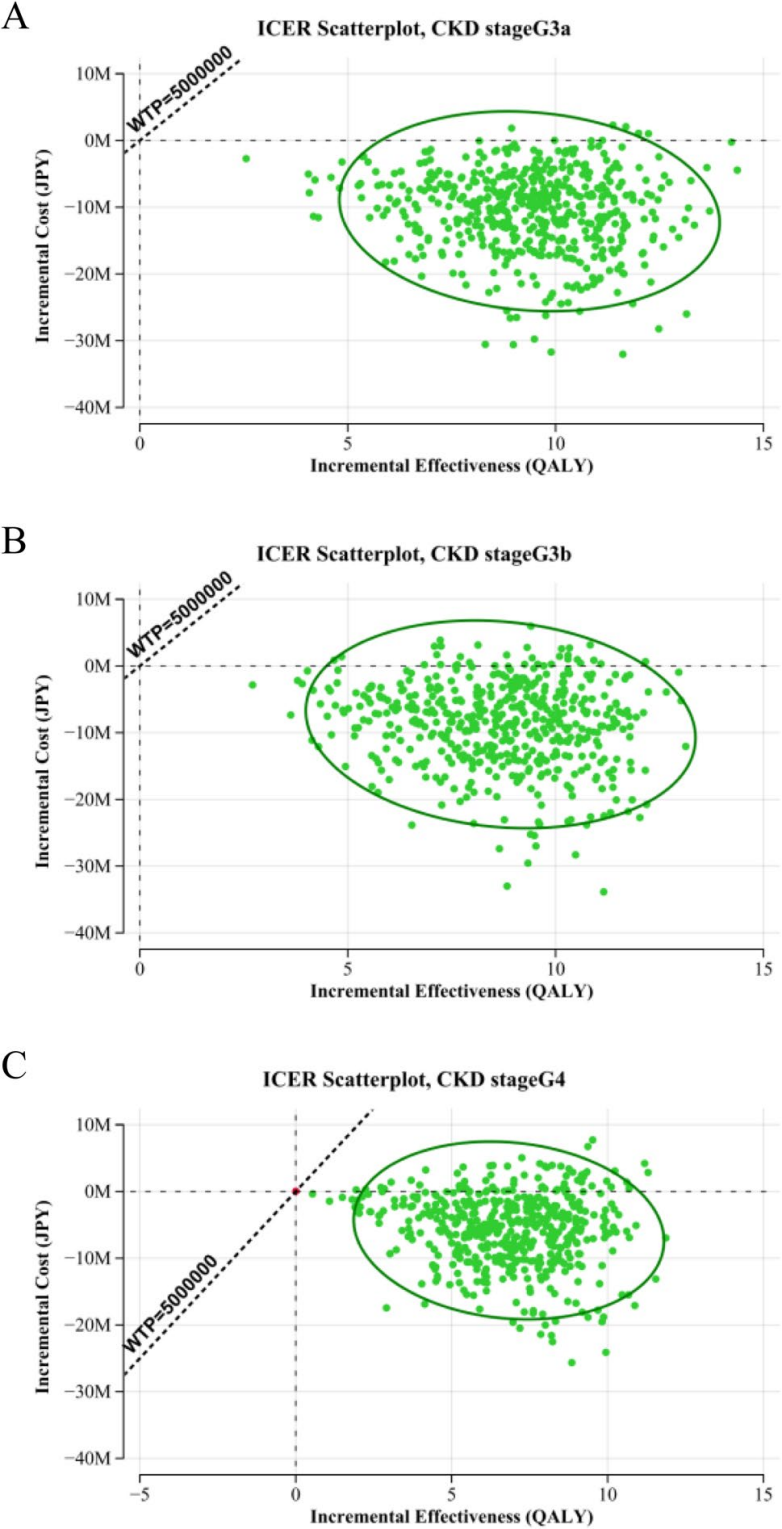
Probabilistic sensitivity analysis: scatterplots. ICER scatterplots of 500 samples for multidisciplinary care over standard treatment. Each dot shows a mean value of 5000 simulations. Ellipses show 95% confidence intervals. Dotted lines show the willingness to pay with a slope of 5 million JPY/QALY. The red dot distributed above the dotted lines indicates a poor cost-effectiveness, and the green dots distributed below the dotted lines indicate good cost-effectiveness. **A**–**C** Patients who were initially at CKD stages G3a, G3b, and G4, respectively. ICER, incremental cost-effectiveness ratio; WTP, willingness to pay; JPY, Japanese yen; QALY, quality-adjusted life-year

Figure 3 shows the scatterplots of ICER of adding MDC to standard treatment. In all the cohorts, nearly all cases were plotted below the line for WTP threshold of 5 million JPY per QALY, indicating good cost-effectiveness of MDC. Furthermore, most of them were plotted at the fourth quadrant (positive incremental QALY and negative incremental cost), indicating that adding MDC was a dominant strategy over standard treatment alone.

**Fig. 4:**
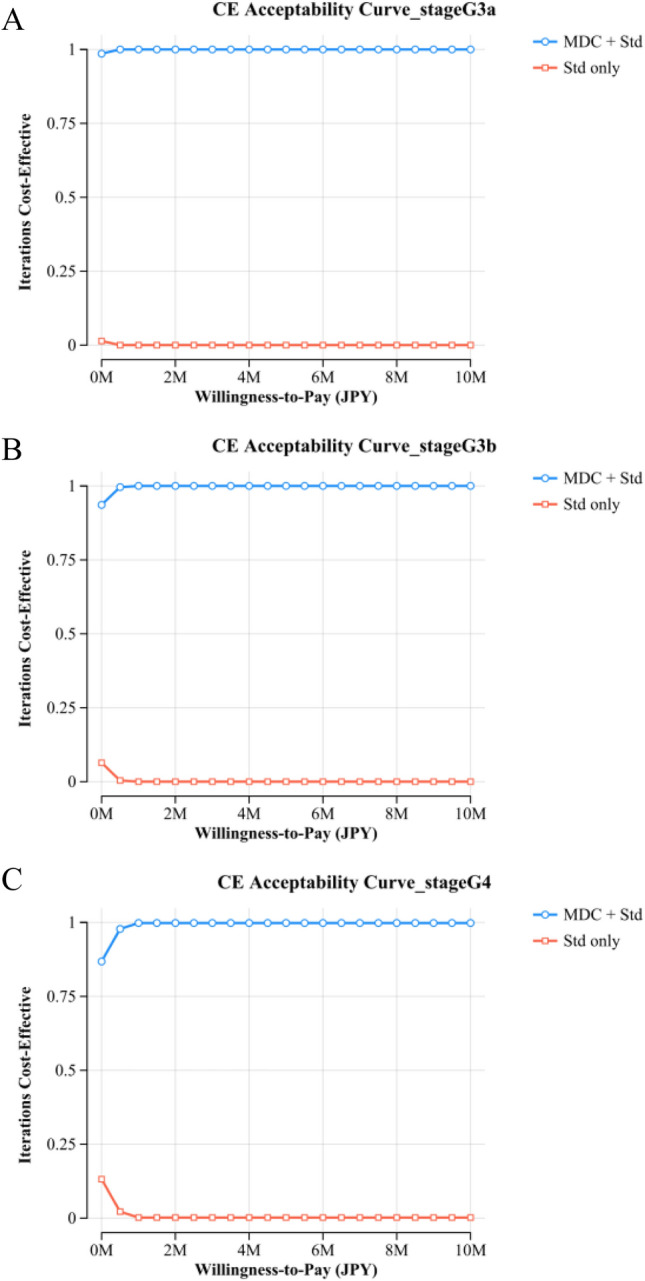
Probabilistic sensitivity analysis: acceptability curves. Cost-effectiveness acceptability curves of multidisciplinary care over standard treatment. Blue lines show the change in proportion of sampling cases that had a good cost-effectiveness judged by each threshold of willingness to pay. **A**–**C** Patients who were initially at CKD stages G3a, G3b, and G4, respectively. MDC, multidisciplinary care; Std, standard treatment; JPY, Japanese yen

The acceptability curves for MDC in each CKD stage cohort are shown in Fig. 4. The acceptability of adding MDC over standard treatment were 1, 1, and 0.998 in CKD stages G3a, G3b, and G4, respectively, at the WTP threshold of 5 million JPY.

### Scenario analysis

The results of scenario analysis are presented in Table 3. A more conservative analysis estimating smaller therapeutic effects demonstrated that MDC remained dominant across all analyzed CKD stages. However, reduced costs and incremental effectiveness were found to be smaller compared to the base-case analysis.

**Table 3:** Scenario analysis

Cohort	Cost (JPY/USD)	QALY	ICER
CKD stage G3a			
MDC	¥40.04 million/$267.8 thousand	17.28	MDC dominant
Standard treatment	¥45.12 million/$301.8 thousand	12.87	
CKD stage G3b			
MDC	¥44.75 million/$299.3 thousand	13.32	MDC dominant
Standard treatment	¥48.55 million/$324.7 thousand	10.70	
CKD stage G4			
MDC	¥49.45 million/$330.8 thousand	9.88	MDC dominant
Standard treatment	¥50.41 million/$337.2 thousand	8.54	

*CKD* chronic kidney disease, *MDC* multidisciplinary care, *QALY* quality-adjusted life-year, *ICER* incremental cost-effectiveness ratio, *JPY* Japanese yen, *USD* United States dollar

## Discussion

Adding MDC to standard treatment of CKD in Japan was shown to be cost-effective in CKD stages G3a, G3b, and G4 cohorts. The addition of MDC resulted in reduced total costs and improved QALYs, suggesting that it is a dominant treatment, across all analyzed cohorts. One-way deterministic analyses showed that the cost of treating kidney failure, the mortality of patients with kidney failure, and the costs of CKD stage G4 and G5 contributed the most to the cost-effectiveness of MDC in all analyzed cohorts. Probabilistic sensitivity analyses indicated that adding MDC remained cost-effective across all cohorts even when uncertainty of the variables were taken into consideration. These findings supported the reliability and robustness of the results. The efficacy of MDC was demonstrated across all stages of CKD, and the earlier MDC was introduced, the greater the cost savings and the larger the increase in QALYs were, suggesting that early intervention for CKD patients by a multidisciplinary team is desirable for both patients and the Japanese healthcare economy.

The previous study demonstrated that MDC suppressed the rate of eGFR decline, and this finding was incorporated into the model as the differences in the variables [14]. Therefore, the observed reduction in total costs and increase in QALYs in this study can be attributed to the impact of MDC on the rate of eGFR decline. Suppression in eGFR decline would keep patients at milder CKD stages with relatively lower cost and mortality, and delay progression to KF stage, leading to reduced costs and increased QALY. While the introduction of MDC is related to additional costs, it is expected to significantly suppress the costs associated with KRT, leading to an overall reduction in total costs. Suppressing the progression of CKD to kidney failure and initiation of KRT is most likely to be the critical factor, as demonstrated by the findings of the one-way deterministic analysis which identified costs and mortality of kidney failure as the most influential factors.

Our finding that MDC is a cost-effective approach in a broad range of CKD population is compatible with previous studies [16, 17]. Importantly, multiple studies suggest that net monetary benefit is larger in patients with milder stage of CKD, which is also demonstrated by our results that reduced cost and gained QALY decrease as CKD stage progresses [16, 19]. One study from the U.S. shows that although MDC is cost-effective, it increased medical costs within an acceptable range of ICER [16]. The reason why MDC saved more costs in our analysis than the previous study may be the different patient transition probabilities. In our analysis which is based on Japanese data, mortality is lower compared to studies from western countries. This results in increased number of patients who transition to the KF stage before death, which would enhance the benefit of MDC in preventing CKD progression and initiation of KRT.

In a previous study, about one third of MDC team consisted of 3 or less staffs, while a nutritionist and a physical therapist are desired to be involved to achieve greater effectiveness [14]. However, recruitment of additional staffs is a critical barrier, especially in small clinics where it is difficult to assign nurses with sufficient experience with CKD management or specialists other than doctors and nurses. One approach to overcome this issue may be utilizing local nutritional care stations to have nutritionists visit the clinics or patients’ home to provide patients with guidance on diet, which, however, is still not easy to carry out consistently. MDC could potentially be implemented in rather large medical facilities, but they also face challenges such as conflicting work schedules among specialists, a lack of space for patient education, and patients’ reluctance to stay long in outpatient clinics.

The strength of our study is that all variable values were based on research conducted in Japan, so we were able to reliably evaluate cost-effectiveness of MDC within the Japanese healthcare system. To the best of our knowledge, no studies have evaluated its cost-effectiveness in Japan.

This study has several limitations. First, although the previous study we referred to for the effects of MDC interventions had a large sample size, it was a retrospective study and followed patients for only a short period of time [14]. Our analysis was based on a premise that the effect of MDC prolongs for a longer time period, which may have overestimated the effect of MDC. The sensitivity analyses showed high robustness of the results even when its uncertainties were considered; however, further research is needed to determine the effect of MDC over a longer term. Additionally, our analysis evaluated the cost-effectiveness of MDC in all CKD stage G3a populations, while Japanese reimbursement for the stage is limited to category A3 [14]. It is not clear whether the benefit of MDC depends on albuminuria or proteinuria, but only results from stage G3aA3 should ideally be incorporated in the analysis, which unfortunately is not available. Second, this model incorporated only two events, kidney failure and all-cause death, and did not take other events, such as cardiovascular events or other hospitalizations, into consideration. This is because whether MDC reduces secondary events associated with CKD in Japan has not been demonstrated. Third, the studies that were referred to for the values of mortality rates, costs, and utilities were published before novel medications, such as SGLT2i, nonsteroidal mineralocorticoid receptor antagonists and glucagon-like peptide-1 receptor agonists, were introduced as standard treatment, whose effects were not considered in our analysis [23, 30–35]. Whether MDC exerts a protective effect on kidney function in addition to such drugs, especially SGLT2i which is now a standard treatment, is uncertain and yet to be clarified in further studies. It is expected that the beneficial effect of MDC is decreased in this background since the baseline decline rate of eGFR would be smaller, which would weaken the cost-effectiveness of MDC. Fourth, compared to other countries, mortality rate in the kidney failure stage is lower and QOL is higher in Japan [30, 34, 35], as described previously. These would lead to underestimation of effectiveness of MDC since it suppresses transition of patients to the stage. However, uncertainty of these variables did not significantly affect the cost-effectiveness in the sensitivity analyses. Also, the mortality in our analysis was not age-adjusted, which could result in longer survival of the population and overestimation of QALY. In particular, incremental survival and QALY may have been overestimated in the MDC group compared to the standard treatment group. The impact of such effect is mitigated, however, by annual discount. Fifth, the cost and the effectiveness were calculated in terms of the Japanese insurance system and healthcare structure in the present analysis. These results cannot be applied directly to other countries or regions. Future research using data from other regions is necessary to further evaluate the global cost-effectiveness of MDC. Also, our analysis applied the reimbursement cost for MDC care in an outpatient setting, while both inpatients and outpatients were evaluated in mixture in the referred study for its effectiveness [14]. The benefit of MDC in suppressing eGFR decrease may differ between these different settings, although it has not been evaluated. However, the components of MDC team are generally consistent between inpatient and outpatient setting in the study, which makes it reasonable to apply its results in our analysis [14].

In conclusion, adding MDC to standard treatment in Japanese patients with CKD is judged as cost-effective in patients with CKD stages G3a, G3b, and G4 based on our model assumptions. MDC is one of the effective non-pharmacological therapies, which is not only clinically effective but also generally free of adverse effects and non-invasive for patients. This study demonstrated its value from a health economics perspective as well.
